# Diabetes and the socioeconomic and built environment: geovisualization of disease prevalence and potential contextual associations using ring maps

**DOI:** 10.1186/1476-072X-10-18

**Published:** 2011-03-01

**Authors:** John E Stewart, Sarah E Battersby, Ana Lopez-De Fede, Kevin C Remington, James W Hardin, Kathy Mayfield-Smith

**Affiliations:** 1 Institute for Families in Society, University of South Carolina, Columbia, South Carolina, USA; 2 Department of Geography, College of Arts and Sciences, University of South Carolina, Columbia, South Carolina, USA; 3 Department of Epidemiology and Biostatistics, Arnold School of Public Health, University of South Carolina, Columbia, South Carolina, USA

## Abstract

**Background:**

Efforts to stem the diabetes epidemic in the United States and other countries must take into account a complex array of individual, social, economic, and built environmental factors. Increasingly, scientists use information visualization tools to "make sense" of large multivariate data sets. Recently, ring map visualization has been explored as a means of depicting spatially referenced, multivariate data in a single information graphic. A ring map shows multiple attribute data sets as separate rings of information surrounding a base map of a particular geographic region of interest. In this study, ring maps were used to evaluate diabetes prevalence among adult South Carolina Medicaid recipients. In particular, county-level ring maps were used to evaluate disparities in diabetes prevalence among adult African Americans and Whites and to explore potential county-level associations between diabetes prevalence among adult African Americans and five measures of the socioeconomic and built environment—persistent poverty, unemployment, rurality, number of fast food restaurants per capita, and number of convenience stores per capita. Although Medicaid pays for the health care of approximately 15 percent of all diabetics, few studies have examined diabetes in adult Medicaid recipients at the county level. The present study thus addresses a critical information gap, while illustrating the utility of ring maps in multivariate investigations of population health and environmental context.

**Results:**

Ring maps showed substantial racial disparity in diabetes prevalence among adult Medicaid recipients and suggested an association between adult African American diabetes prevalence and rurality. Rurality was significantly positively associated with diabetes prevalence among adult African American Medicaid recipients in a multivariate statistical model.

**Conclusions:**

Efforts to reduce diabetes among adult African American Medicaid recipients must extend to rural African Americans. Ring maps can be used to integrate diverse data sets, explore attribute associations, and achieve insights critical to the promotion of population health.

## Background

Nearly 24 million people in the United States—approximately 8 percent of the population—have diabetes [1], a serious disease that can result in blindness, kidney failure, peripheral neuropathy and arterial disease, cognitive impairment, and death [2]. An estimated one-fourth of all diabetes cases are undiagnosed [1]. Ninety to 95 percent of diagnosed cases are the non-insulin-dependent type 2 form of the disease, characterized by insulin resistance, or the inability of cells to effectively use insulin; the next largest group of diabetics have the type 1 or juvenile-onset form of the disease, characterized by the body's inability to make insulin [1,2]. Older persons are particularly at risk for diabetes. Indeed, the prevalence of diagnosed diabetes in persons 65 years and older is more than 12 times the rate of that among persons 44 years and younger [3]. Diabetes prevalence in younger persons, however, is on the rise. Among individuals aged 30 to 39 years, for instance, the prevalence of diabetes rose from 1.4 percent in 1991 to 3.7 percent in 2001. By 2021, 5.0 percent of individuals in this age group are expected to have the disease [4]. Increasing diabetes prevalence among younger adults suggests that the burden of this disease on population health, and on the nation's health care system, will only worsen in years to come.

In addition to age, known individual-level risk factors for diabetes include genetic predisposition, race/ethnicity (diabetes prevalence is approximately 50 percent higher in African American adults relative to White adults), increased body mass index (BMI), and physical inactivity [1,5,6]. Moreover, a growing body of evidence suggests an association between diabetes, diabetes-related conditions (e.g., cardiovascular disease and obesity) and characteristics of the socioeconomic and built environment. Studies have found, for example, a higher incidence of diabetes among African American women in low socioeconomic status (SES) versus higher SES neighborhoods [7], greater risk of coronary heart disease in socioeconomically disadvantaged versus more affluent census block groups [8], increased BMI among women in areas of high unemployment relative to areas of low unemployment [9], and higher rates of obesity in socioeconomically deprived neighborhoods compared to more affluent neighborhoods [10] (readers are directed to the individual studies cited for detailed information on the definition of small area socioeconomic position used in each respective study). Other investigations indicate higher rates of diabetes [11,12] and obesity [11,13,14] in rural areas relative to urban centers. Poorer health status in socioeconomically deprived and rural environments may reflect, in part, the inaccessibility of such built environmental features as public pools, recreation centers, physical fitness facilities, parks, sidewalks, and streetlights [15-17]. The availability and quality of local food establishments also may be associated with health status. For example, lower rates of obesity have been noted among persons living in census tracts with large chain supermarkets (which sell a wide assortment of food products, including fresh fruits and vegetables), while higher rates of obesity have been found among residents of census tracts with convenience stores (which typically offer a limited selection of foodstuffs and little, if any, fresh produce) [18].

Efforts to stem the diabetes epidemic in the Unites States [2,19,20] must take into account a complex array of individual, social, economic, and built environmental factors. In an age characterized by ever mounting volumes of data, scientists increasingly use information visualization tools to "make sense" of data inputs, to discover critical patterns and associations, restructure problems, and achieve insight through new perspectives [21]. Spatial visualization tools—typically maps—are widely used in the communication of geographic data. The presentation of multivariate data in maps, however, can present numerous cartographic challenges related to classification, symbolization, legibility, and interpretation. For representation of three or more attributes, small multiples—a set of small maps depicting related attributes—can be used to facilitate the evaluation of complex, multivariate spatial patterns [22]. Although small multiples aid in the visualization of patterns, the ability to interpret and integrate information across small multiple maps can prove difficult [23], particularly if an individual is attempting comparisons between specific locations or between complex attributes [24].

More recently, ring map visualization has been explored as a means of depicting spatially referenced, multivariate data in a single information graphic. A ring map depicts multiple attribute data sets as separate rings of information surrounding a base map of a particular geographic region of interest [25,26]. Multivariate ring maps have been used to evaluate spatio-temporal patterns of human activity [26], track pandemic H1N1 infection [27], and assess potential small area associations between socioeconomic disadvantage and HIV/AIDS [28].

Diabetes prevalence rates in South Carolina are among the highest in the nation [29]. In this study, ring maps were used to evaluate diabetes prevalence among adult South Carolina Medicaid recipients. In particular, county-level ring maps were used to highlight disparities in diabetes prevalence among adult African Americans and Whites and to explore potential county-level associations between diabetes prevalence among adult African Americans and five measures of the socioeconomic and built environment—persistent poverty, unemployment, rurality, number of fast food restaurants per capita, and number of convenience stores per capita. Although Medicaid pays for the health care of approximately 15 percent of all diabetics [30], few studies have examined diabetes in adult Medicaid recipients at the county level. The present study thus addresses a critical information gap, while illustrating the utility of ring maps in multivariate investigations of population health and the socioeconomic and built environment.

## Methods

### Data

Diabetes prevalence was calculated for adults aged 18 and older enrolled in South Carolina Medicaid between July 2008 and June 2009 (N = 442,830) [31]. Medicaid enrollees were identified as having diabetes if paid claims indicated an ICD-9 diagnosis of 250.0 through 250.9. The use of administrative paid claims data (as opposed to clinical records) in this study did not allow the differentiation of type 1 and type 2 diabetes (women with gestational diabetes were excluded) and did not capture diabetics not yet diagnosed with the disease.

County-level unadjusted diabetes prevalence rates (per 100) were calculated by dividing the number of adult Medicaid recipients with diabetes by the total number of adult Medicaid participants in each of the state's 46 counties and by multiplying the resulting values by 100. Separate unadjusted diabetes prevalence rates were calculated for African American and White adults (N = 202,263 and N = 195,588, respectively) to examine racial differences in diabetes at the county level. Age-adjusted prevalence rates were used in a subsequent evaluation of potential associations between environmental context and diabetes in African American Medicaid recipients aged 18 and older to account for county-level variation in age structure among African Americans. Rates were standardized based on the U.S. population of non-Hispanic Blacks aged 18 and older in the year 2000 [32]. Age-standardized rates and 95-percent confidence intervals were calculated using methods described by Curtin and Klein (1995) [33].

Five county-level environmental measures were used in the evaluation of potential contextual associations with diabetes in African Americans aged 18 and older. Area-level indices of socioeconomic disadvantage included a binary (present/absent) measure of persistent poverty (20 percent or more of residents falling below the federal poverty level in each of the decennial census years 1970, 1980, 1990, and 2000) [34], and an unemployment index (percent of the civilian labor force without jobs) [35]. Rurality was defined as the percent of county residents living in rural areas [32]. Built environmental measures included the number of chain fast food restaurants per capita, and the number of convenience stores per capita. Chain fast food and convenience store establishments were identified using Standard Industrial Classification (SIC) codes and an electronic business directory [36] (chain fast food restaurants = SIC code 58120307; convenience stores = SIC codes 54110200, 54110201, and 54110202). A geographic information system was employed to spatially locate fast food and convenience store establishments and to summarize the number of each establishment type by county [37]. Per capita rates were calculated using 2008 county population estimates [38]. Based on results from previous studies [7,11,12,18,39], a positive association was expected between diabetes prevalence among adult African American Medicaid recipients and each of the five environmental measures.

### Ring Map Development

**Figure 1:**
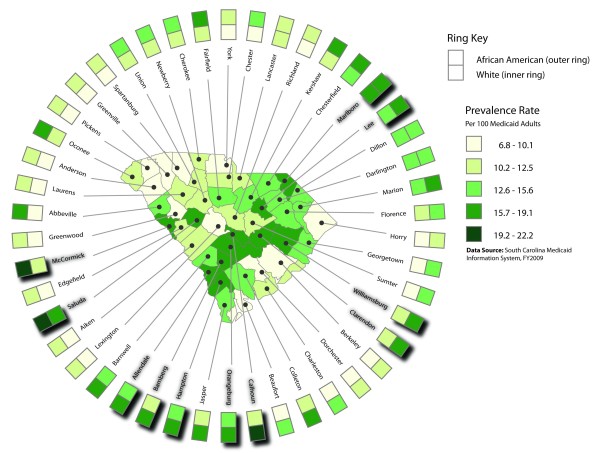
**Unadjusted diabetes prevalence rates for all adult Medicaid recipients (base map) and race specific rates (rings)**. The base map in the center of the figure shows unadjusted diabetes prevalence rates for all South Carolina adult Medicaid recipients at the county level. The 46 spokes surrounding the base map represent each of the state's 46 counties. Extending outward from the central base map, each spoke consists of a leader line connecting a specific county on the base map with its county label, a square polygon showing county-level unadjusted diabetes prevalence among White adult Medicaid recipients, and a square polygon showing county-level unadjusted diabetes prevalence among African American adult Medicaid recipients. The set of 46 polygons showing diabetes prevalence among Whites forms the innermost ring of the ring map, while the 46 polygons showing diabetes prevalence among African Americans form the outermost ring of the map. The prevalence rates shown in the legend encompass the entire range of rates for all adult Medicaid recipients, African American adult Medicaid recipients, and White adult Medicaid recipients. Shadow effects underneath some county labels and square polygons serve to highlight those counties in the highest quartile of the county-level unadjusted diabetes prevalence rate distribution for all adult Medicaid recipients.

County-level ring maps were designed with attention paid to similar techniques employed by Huang, et al. (2008) [25] and Zhao, et al (2008) [26]. Modifications were made to align the method with the multivariate data representation needs of the present study. To create a ring map, a core circle area large enough to contain a base map of South Carolina counties was established (Figure 1). A set of attribute "spokes" was then drawn, with spokes distributed evenly in a radiating fashion around the base map. Each spoke on the ring map presents one or more attributes for a single county. A set of attribute-specific spoke elements forms a "ring" of information around the core. Attribute data contained in rings are spatially referenced through the use of leader lines that tie each spoke to its respective county representation on the base map. The choroplethic base map, itself, presents an additional data layer (Figure 1). Ring maps were created in Adobe Illustrator [40] through the application of a custom script that dynamically drew, distributed, and symbolized all graphic map elements. The values for symbolization were read from a Comma Separated Value (CSV) file that contained all county attribute data.

**Figure 2:**
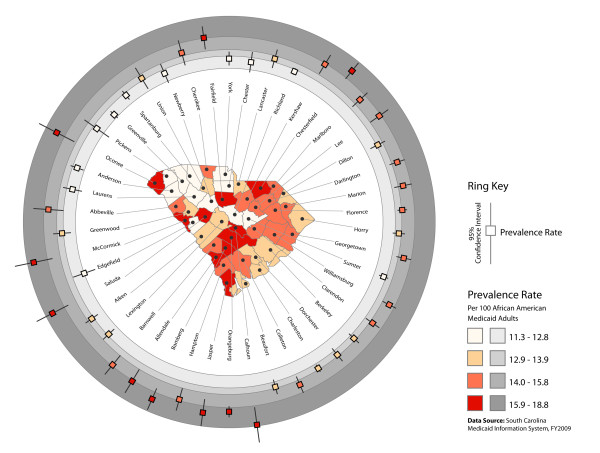
**Age-adjusted diabetes prevalence among adult African American Medicaid recipients (base map) with associated 95 percent confidence intervals (rings)**. The base map in the center of the figure shows age-adjusted diabetes prevalence rates for adult African American Medicaid recipients at the county level. Prevalence rate classes represent quartiles of the county-level prevalence rate distribution. Prevalence rate quartiles are symbolized using an orange color ramp in the base map, and for graphic differentiation a gray color ramp in the rings surrounding the base map. Superimposed on the rings, a set of 95 percent confidence interval symbols, each consisting of a central square and two extending "arms," shows the age-adjusted diabetes prevalence rate (square) and the 95 percent confidence interval associated with that rate for each county. The ring map differentiates counties with relatively wide versus narrow 95 percent confidence intervals (e.g., Oconee versus Anderson in the northwest portion of the state), highlights instances in which the 95 percent confidence interval extends through multiple quartile ranges (e.g., Cherokee in the northern part of the state), and shows where the 95 percent confidence intervals of counties grouped in different quartiles overlap (e.g., Abbeville and Greenwood in the western part of the state).

**Figure 3:**
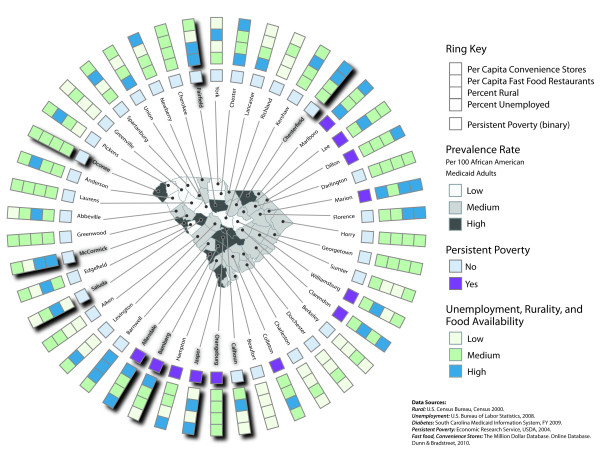
**Age-adjusted diabetes prevalence among adult African American Medicaid recipients (base map) and environmental characteristics (rings)**. For the ordered category variables–diabetes prevalence rate, unemployment, rurality, fast food restaurant availability, and convenience store availability–"Low" equals the bottom quartile, "Medium" equals the second and third quartiles combined, and "High" equals the top quartile of the respective data distributions. Shadow effects underneath some county labels and square polygons serve to highlight those counties in the highest quartile of the county-level age-adjusted diabetes prevalence rate distribution for African American adult Medicaid recipients.

Three county-level ring maps were developed. The first map (Figure 1) shows the unadjusted diabetes prevalence rate among all South Carolina Medicaid recipients aged 18 years and older (base map), and among adult African American and adult White Medicaid recipients (rings). The second map (Figure 2) depicts the age-adjusted diabetes prevalence rate for African American Medicaid recipients aged 18 years and older (base map), with 95-percent confidence intervals associated with age-adjusted prevalence estimates shown relative to prevalence rate distribution quartile ranges (rings). Finally, the third map (Figure 3) shows relative levels (high, medium, and low) of age-adjusted diabetes prevalence among adult African American Medicaid enrollees (base map); the presence or absence of persistent poverty (innermost ring); and relative levels of unemployment, rurality, fast food restaurant availability, and convenience store availability (outer rings). Relative attribute levels were established based on quartile rankings for each county-level attribute distribution (high = top quartile, medium = second and third quartiles, low = bottom quartile).

### Statistical Methods

Statistical analyses were conducted to quantitatively evaluate potential county-level associations between diabetes prevalence in adult African American Medicaid recipients and each of the five environmental predictor variables. In all analyses, diabetes prevalence rates were grouped into three ordered categories—high, medium, and low—with high representing the top quartile of the county prevalence rate distribution, medium representing the second and third quartiles, and low representing the bottom quartile. Persistent poverty was evaluated as a binary variable (present or absent). The four remaining environmental predictors also were grouped into three ordered categories, based on quartile rankings for each county-level attribute distribution (high = top quartile, medium = second and third quartiles, low = bottom quartile). Both bivariate and multivariate ordered logistic regression models were tested. Due to the geographic nature of the data, geographically weighted analyses also were conducted. These tests yielded insufficient evidence that geographic weights improved the global (unweighted) analyses. The results reported, therefore, are from the simpler unweighted models. All statistical analyses were conducted using Stata version 11.0 [41].

## Results

The diabetes prevalence rate among South Carolina Medicaid recipients aged 18 and older was 11.6 percent. Among subjects younger than 45 years, the rate was 3.9 percent, versus 21.5 percent among those 60 years of age and older. County-level diabetes prevalence rates ranged from 9.0 percent to 19.1 percent (mean = 13.1, SD = 3.0). Relatively high rates of diabetes existed in inland counties, especially along a transect extending from Allendale in the southwestern portion of the state to Marlboro in the northeast (Figure 1). The diabetes prevalence rate was 13.8 percent among adult African American Medicaid recipients, versus 9.6 percent among Whites. County-level rates ranged from 10.6 percent to 22.2 percent for African Americans (mean = 14.9, SD = 2.9) and from 6.8 percent to 16.1 percent for Whites (mean = 10.9, SD = 2.4). County-level rates for African Americans were higher than those for Whites in 45 of the state's 46 counties. Figure 1 shows that in 16 counties diabetes rates among African Americans were two or more class intervals higher than rates among Whites (contrast inner and outer rings). In three counties, diabetes prevalence among African Americans exceeded 19.1 percent (Figure 1).

County-level age-adjusted rates for adult African American Medicaid recipients ranged from 11.3 percent to 18.8 percent. Ninety-five percent confidence intervals associated with age-adjusted prevalence estimates reflected relative population size, with the widest intervals occurring in counties with the smallest number of adult African American Medicaid participants (Figure 2).

A visual association was apparent between diabetes prevalence among adult African Americans and the relative rurality of counties (Figure 3). Five counties in the highest diabetes prevalence quartile (dark gray in the figure) also were in the highest rurality quartile (dark blue in the figure) and none were in the lowest rurality quartile (very light green in the figure). Conversely, five counties in the lowest diabetes prevalence quartile (white in the figure) also were in the lowest rurality quartile; one, however, was in the highest rurality quartile. A visual association also existed between African American diabetes prevalence and persistent poverty. Four counties in the highest diabetes prevalence quartile were persistent poverty counties (purple in the figure), while none of the counties in the lowest diabetes prevalence quartile was persistently impoverished. Less visual correlation was apparent between African American diabetes prevalence and unemployment, number of fast food stores per capita, and number of convenience stores per capita (Figure 3).

Bivariate statistical analyses indicated a strong positive association between rurality and diabetes prevalence (odds ratio = 3.6, p-value = 0.005). Living in a persistent poverty county also was positively associated with diabetes prevalence (odds ratio = 3.9, p-value = 0.040). In a multivariate generalized ordered logistic regression model, rurality was significantly positively associated with diabetes prevalence among adult African American Medicaid recipients (odds ratio = 3.1, p-value = 0.018); however, after adjusting for rurality, living in a persistent poverty county was no longer significantly associated with diabetes prevalence (Table 1).

**Table 1:** County-level association between age-adjusted diabetes prevalence in adult African American Medicaid recipients and environmental characteristics

Environmental Characteristic	Statistical Model	
	Bivariate-Ordered Category	Multivariate-Ordered Category
		
Persistent Poverty	OR = 3.9	OR = 2.5
	95% CI [1.06-14.53]	95% CI [0.63-10.10]
	P = 0.040	P = 0.191
		
Unemployment	OR = 1.7	
	95% CI [0.75-4.03]	
	P = 0.198	
		
Rurality	OR = 3.6	OR = 3.1
	95% CI [1.47-8.76]	95% CI [1.21-7.83]
	P = 0.005	P = 0.018
		
Fast Food	OR = 0.4	
Restaurants	95% CI [0.09-1.41]	
Per Capita	P = 0.140	
		
Convenience	OR = 1.5	
Stores	95% CI [0.41-5.63]	
Per Capita	P = 0.526	

The multivariate model included only those predictor variables that were significant in bivariate models at the .05 alpha level. OR = odds ratio.

## Discussion and Conclusions

Medicaid claims data are collected for administrative rather than research purposes. The use of administrative claims codes, which reflect both covered services and reimbursement practices, may result in under- or over-estimates of the prevalence of diabetes in the study population. A broad range of diabetes diagnostic codes encompassing both type 1 and type 2 diabetes was used to define the eligible population. The contextual associations found in this study may vary by diabetes diagnosis. Future research would benefit from the use of clinical records to define diabetes by type and to account for different type-specific physical severity levels.

The mechanisms by which area-level socioeconomic disadvantage adversely affects health are complex and incompletely understood. Small-area socioeconomic deprivation, itself, may directly compromise individual health; alternatively, relatively poor health outcomes in socioeconomically disadvantaged areas may reflect reduced access to health care, limited social support, social disorder, exposure to hazardous environmental pollutants, and/or local discriminatory practices [42-44]. In this study, both county-level measures of socioeconomic disadvantage—persistent poverty and unemployment—were positively associated with diabetes prevalence among adult African American Medicaid recipients in bivariate models. Of the two measures, however, only persistent poverty was a significant bivariate predictor. The lack of a statistically significant association between persistent poverty and diabetes in the multivariate model may be due in part to correlation of the predictor variables (although model diagnostics indicated no collinearity). Additional studies are needed to clarify the impact of persistent poverty at the small area level on diabetes prevalence.

Rurality was significantly positively associated with diabetes prevalence in both bivariate and multivariate models. Compared to urban counties, primarily or completely rural counties had higher rates of diabetes among adult African American subjects, even after adjusting for local levels of unemployment, persistent poverty, and food establishment availability. These results are consistent with other investigations showing higher rates of diabetes in rural versus urban areas [11,12]. Elevated rates of diabetes in rural regions might reflect diminished access to primary care [45], a lack of sidewalks or other safe places to walk [16,17,46], the relative inaccessibility of parks and recreational facilities [17,46], low social support [17], and/or regionally-specific rural cultural norms that can undermine health [46,47]. The observed association between diabetes prevalence and rurality has important implications for public health policy creation and health promotion planning. In particular, efforts to reduce the burden of diabetes among adult African Americans must extend beyond city boundaries and address in culturally relevant ways the specific health needs of rural African Americans in South Carolina and across the Southern "black belt [48]." Notably, the association between rurality and diabetes in this study was specific to adult African American Medicaid recipients and should not be generalized to non-African American or younger Medicaid recipients, or to the broader non-Medicaid population.

Neither measure of the built environment was significantly associated with diabetes prevalence. This result might partly reflect the study's reliance on a single electronic business directory to identify and spatially locate chain fast food restaurants and convenience stores. Like other commercial business directories, the Dunn and Bradstreet product used in this investigation may contain incomplete business listings, outdated information, and/or street address data for corporate headquarters rather than local places of business. In addition, the self-classification of business type in the Dunn and Bradstreet directory may lead to inconsistent classification across listings. Further investigations are needed to evaluate potential small-area associations between diabetes and the relative availability of unhealthy food outlets, the proximity of such food establishments (e.g., distance to the nearest fast food restaurant or convenience store) [49], and the frequency with which fast food or convenience-type food products are consumed [39]. Future studies also might consider potential contextual associations between diabetes and access to such healthy food outlets as "green" or farmers' markets, roadside fruit and vegetable stands, and large supermarkets with extensive produce sections.

Recognition of the dependent relationship between spatial phenomenon and geographic unit of analysis—the so-called Modifiable Areal Unit Problem—is critical to the understanding of studies that employ spatially aggregated data [50]. In this investigation, diabetes prevalence and environmental data were aggregated at the county level. The results obtained, therefore, reflect only county-level environmental influences on diabetes prevalence among adult African Americans. Associations between diabetes and environmental context may be different at larger (e.g., state or national) and smaller (e.g., census tract or census block group) geographic scales. County-level spatial analyses of health are appropriate when county-level agents (e.g., county health departments, park and recreation departments, regulatory commissions, and councils on aging) play direct roles in disease prevention/intervention and wellness promotion programs. Although county-level investigations may suggest potential associations between health and environment at different geographic scales, such associations must be evaluated separately using appropriate geographic units of analysis.

As this study shows, ring maps can highlight racial disparities in health, convey epidemiological uncertainty data (e.g., confidence interval data associated with standardized morbidity and mortality rates), and suggest small area-level associations between adverse health outcomes and characteristics of the socioeconomic and built environment. The ring maps presented here only begin to illustrate the potential utility of this visualization method for health geographers. For example, ring maps can depict multiple attributes at the census tract, census block group, ZIP code area, hospital catchment area, or public health service area level, in addition to the county level as shown in the figures [25,27]. In addition, a ring map can be used to depict a single attribute at multiple geographic scales. For instance, a ring map might show diabetes prevalence rates for South Carolina at the census tract level in a base map and prevalence rates at the county level and public health region (multiple county) level in successive rings (in this case, the number of enumeration units in the inner ring would reflect the number of counties, and the number of enumerations units in the outer ring would reflect the number of health regions in the state). Ring maps also can display time-series data for a single variable of interest [25,27]. A map with six rings might show annual incidence rates of cardiovascular disease over a six-year period, for example; alternatively, a ring map might depict the weekly incidence of cases associated with an influenza outbreak. Ring maps thus permit the exploration of relevant distributions, patterns, and associations across both space and time [25,27]. Additional layers of data are easy to add to ring map visualizations, requiring only that new rings be drawn [27]. Although the ring maps shown are circular, elliptical or even non-continuous rings can be drawn to accommodate irregularly shaped geographic regions [25,27]. In short, ring maps provide sufficient flexibility in design and development to permit the visualization—and visual exploration—of spatiotemporal data across a wide range of health applications.

A distinct problem associated with ring map visualization is the loss of complete topology (i.e., information about the spatial relationships of geographic units) in the rings. Although a single county in South Carolina may have as many as nine adjacent neighbors, only two adjacent neighbors (spokes) exist in ring displays. Complete spatial topology is retained in the central base map, though, allowing users to determine adjacent relationships, relative direction, and the relative nearness or farness of geographic units. Another practical limitation is the ability of ring maps to display data for a large number of geographic units. It would be challenging to construct—and difficult to interpret—a ring map depicting the more than 800 census tracts in South Carolina, for instance. Ring maps—as they appear in print—also suffer limitations common to all static map products. They depict a set number of predetermined data layers, using a static data classification method, and an unchanging symbolization scheme.

Most of the graphic limitations associated with static ring map visualization, including the limited representation of spatial topology in rings, might be addressed in a dynamic ring mapping environment. In such an environment, a user could interactively select the geographic area of interest, establish the geographic scale of representation, choose data elements for exploration, assign attributes to rings, modify the classification and symbolization of data, reorder (and even resize and reshape) rings, and "click on" one or more ring attribute elements to see the corresponding geographic unit(s) highlighted on the base map in complete spatial topological context. A dynamic and fully interactive ring mapping application of this sort would represent a powerful information visualization tool with which to integrate and explore diverse data sets, frame questions, generate hypotheses, restructure problems, and achieve insights [21] critical to the protection and promotion of population health.

Further studies are needed to evaluate the relative strengths and weaknesses of ring maps—in both static and dynamic forms—as multivariate data visualization tools. Such studies might explore the optimal number of rings to display for specific data types and data type combinations, the maximum number of aggregation units that can be effectively depicted, and the effect of color symbolization on ring map interpretation. Finally, research is needed to demonstrate the relative effectiveness of ring maps versus small multiple map displays in the visualization of multivariate health data.

## Competing interests

The authors declare that they have no competing interests.

## Authors' contributions

JES co-conceived and coordinated the study, helped derive the data, helped develop the ring maps, and drafted the manuscript. SEB co-conceived the study, developed the ring maps, and helped to draft the manuscript. ALD co-conceived the study, provided all Medicaid data, and helped to draft the manuscript. KCR co-conceived the study, performed GIS analyses, helped derive the data, helped develop the ring maps, and helped to draft the manuscript. JWH performed all statistical analyses and helped to draft the manuscript. KMS co-conceived the study and contributed to the manuscript. All authors read and approved the final manuscript.
